# Socio-Demographic Disparities in Gastric Adenocarcinoma: A Population-Based Study

**DOI:** 10.3390/cancers12010157

**Published:** 2020-01-09

**Authors:** Navpreet Rana, Rohit Gosain, Riccardo Lemini, Chong Wang, Emmanuel Gabriel, Turab Mohammed, Beas Siromoni, Sarbajit Mukherjee

**Affiliations:** 1 Department of Medicine, University at Buffalo School of Medicine, Buffalo, NY 14263, USA; 2 Division of Hematology & Oncology, Roswell Park Comprehensive Cancer Center, University at Buffalo School of Medicine, Buffalo, NY 14263, USA; 3 Department of Surgical Oncology, Mayo Clinic, Rochester, MN 55905, USA; 4 Department of Medicine, University of Connecticut School of Medicine, Farmington, CT 06030, USA; 5 Department of Medicine, University of Connecticut Health, Hartford, CT 06030, USA; 6 Institute of Agricultural Sciences, University of Calcutta, West Bengal 700073, India

**Keywords:** gastric, disparities, rural, urban, incidence, outcomes, adenocarcinoma, stomach

## Abstract

**Background:** Gastric cancer is one of the leading causes of cancer-related mortality worldwide, accounting for 8.2% of cancer-related deaths. The purpose of this study was to investigate the geographic and sociodemographic disparities in gastric adenocarcinoma patients. Methods: We conducted a retrospective study in gastric adenocarcinoma patients between 2004 and 2013. Data were obtained from the National Cancer Data Base (NCDB). Univariate and multivariable analyses were performed to evaluate overall survival (OS). Socio-demographic factors, including the location of residence [metro area (MA) or rural area (RA)], gender, race, insurance status, and marital status, were analyzed. Results: A total of 88,246 [RA, N = 12,365; MA, N = 75,881] patients were included. Univariate and multivariable analysis showed that RA had worse OS (univariate HR = 1.08, *p* < 0.01; multivariate HR = 1.04; *p* < 0.01) compared to MA. When comparing different racial backgrounds, Native American and African American populations had poorer OS when compared to the white population; however, Asian patients had a better OS (multivariable HR = 0.68, *p* < 0.01). From a quality of care standpoint, MA patients had fewer median days to surgery (28 vs. 33; *p* < 0.01) with fewer positive margins (6.3% vs. 6.9%; *p* < 0.01) when compared to RA patients. When comparing the extent of lymph node dissection, 19.6% of MA patients underwent an extensive dissection (more than or equal to 15 lymph nodes) in comparison to 18.7% patients in RA (*p* = 0.03). Discussion: This study identifies socio-demographic disparities in gastric adenocarcinoma. Future health policy initiatives should focus on equitable allocation of resources to improve the outcomes.

## 1. Introduction

Gastric cancer (GC) is the fifth most common cancer worldwide and the third leading cause of cancer-related death [1]. The molecular biology of GC is complex and varies by site and histology. The vast majority of GC are adenocarcinomas arising from the glands of the superficial layer, or the mucosa of the stomach. In this era of personalized medicine, the management of gastric cancer is becoming increasingly more complex and is often dependent on multidisciplinary involvement. Access to specialized cancer centers is vital, where timely management can be initiated. Unfortunately, socioeconomic barriers often prevent patients from getting access to cancer care. A recent report showed that cancer mortality has been steadily declining for the most common cancers in the US, which was attributed to early detection. The study also showed that although the racial gap in cancer death has been narrowing in the US, socio-economic inequalities are getting wider [2].

Few studies have looked into the potential socio-demographic disparities in gastric adenocarcinoma patients. In a recent study, Jim et al. looked at stomach cancer survival in the United States (U.S.) by race and stage. They reported a 5-year survival rate of 29%, with no differences observed based on race [3]. Extensive research has been conducted using country-specific registries like SEER and institutionally based databases, which have evaluated differences amongst survival and incidence in different countries and people of various racial/ethnical backgrounds [1,4,5,6,7]. The results of these studies have not been uniform. Wang et al. evaluated incidence and prognosis among gastroesophageal cancer patients in rural and urban populations, utilizing the SEER registry; the authors found no differences in prognosis, based on the area of residence [7]. On the other hand, a study from China looked at the disparity among rural versus urban patients with different cancers and found that 5-year observed and relative survivals of liver, pancreas, stomach, brain, and prostate cancer were higher in urban than in rural areas [8]. 

Therefore, to understand the effect of geographic and sociodemographic factors in the gastric cancer population, we conducted a large population-based study utilizing the National Cancer Database (NCDB) from 2004 to 2013. Given that most common histology in gastric cancer patients is gastric adenocarcinoma (more than 90%), we included only those patients in our analysis.

## 2. Materials and Methods

The NCDB is a hospital-based cancer registry sponsored by the American College of Surgeons and the American Cancer Society, which represents 70% of all newly diagnosed cancer cases in the US [8]. Utilizing the NCDB participant user file, a retrospective analysis was performed on patients diagnosed between 2004 and 2013. The histology codes for gastric adenocarcinoma (8140) were based on the ICD-03/WHO 2008 classification. Patients from all stages, as per the American Joint Committee on Cancer (AJCC 6th and 7th edition) guidelines, were included as a part of the analysis. Patients with unknown stage were excluded from the analysis. To control for confounders, the following variables were utilized in the multivariable analyses: age, sex, race, Charlson/Deyo comorbidity score, and tumor grade.

In order to evaluate survival outcomes based on place of residence, we classified patients into urban versus rural. The patients were classified utilizing the Rural–Urban Continuum Code (RUCC), a variable available in the NCDB. The Office of Management and Budget (OMB) classified RUCC into two categories, metropolitan and non-metropolitan areas. A metropolitan was defined to be the area where the population ranged from less or more than 250,000. On the other hand, a non-metropolitan statistical area is comprised of 20,000 or more individuals adjacent to the metro area. To be consistent with other studies, counties classified as metropolitan (RUCCs 1–3) were classified as metro, and counties classified as non-metropolitan (RUCCs 4–9) were classified as rural [9,10].

Using the NCDB, we analyzed the data based on various socio-demographic factors, including age, sex, race, marital status, insurance status, residence (rural or metro), and also tumor-related factors (stage, grade, histology, and primary site). Additionally, we utilized the Surveillance, Epidemiology, and End Results (SEER) database and evaluated data in gastric cancer patients diagnosed between 1973 and 2015. The SEER database collects cancer-specific data from population-based cancer registries. It collects incidence, survival and other essential data regarding patients and their tumors. We utilized the SEER database to report gastric adenocarcinoma incidence and disease-specific survival (DSS) in particular.

Associations between the area of residence and various socio-demographic and tumor-specific characteristics were assessed using the Kruskal–Wallis test (for ordinal responses) or the Pearson Chi-square test (for categorical responses). The primary endpoint of the study was to estimate overall survival (OS), defined by the time from diagnosis of gastric cancer to death due to any cause. To evaluate OS utilizing different covariates, we used univariate and multivariable Cox proportional hazard models, adjusting for age, sex, stage, grade, tumor location, year of diagnosis, insurance status, marital status, race, and area of residence. Result estimates were expressed as hazard ratios (HR) with 95% confidence intervals (CI). Long-term survival was evaluated utilizing the Kaplan–Meier method, with comparisons based on the log-rank test. Baseline characteristics and outcomes were compared between the individual groups. Statistical significance was indicated by *p* values less than 0.05. All statistical analyses were performed using Statistical Analysis Software (SAS version 9.4)

## 3. Results

**Figure 1:**
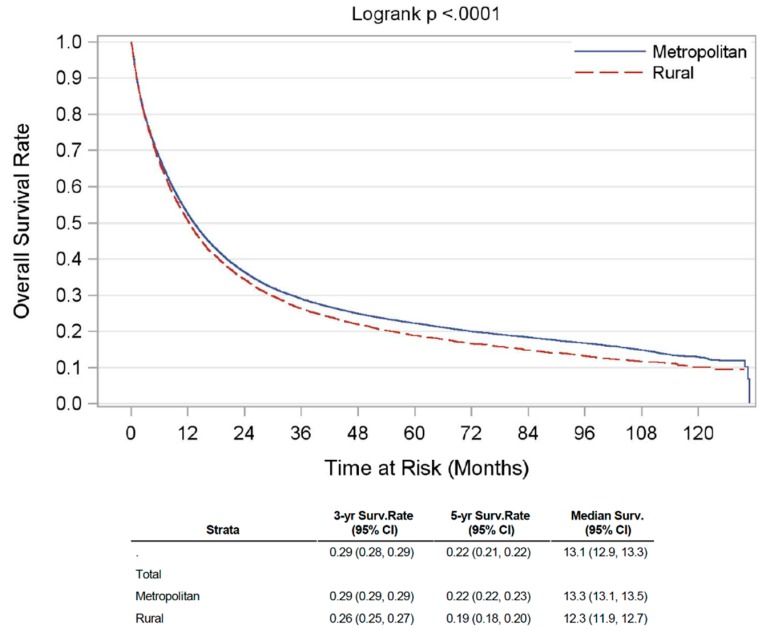
Unadjusted Kaplan–Meier OS curve for gastric adenocarcinoma patients in rural versus metropolitan region.

A total of 88,246 patients with gastric adenocarcinoma were included in the analysis, of whom 12,365 (14%) patients had a residence in rural areas (RA), and 75,881 (86%) individuals resided in metro areas (MA). The median age at diagnosis was 70 years. Individuals in RA had a slightly younger age at presentation compared to their urban counterparts (median 69 years versus 70 years, *p* < 0.01). The urban population had a larger proportion of African American (AA) and Asian patients as compared to the rural population (Asian—7% versus 1%; African-American—16% versus 10%; *p* < 0.01). Patients from MA presented at an earlier stage as compared to those from RA. The primary site of diagnosis in RA was mainly fundus and cardia of the stomach when compared (50% versus 40%; *p* < 0.01) to the MA population. Further comparison of all the baseline characteristics between RA and MA population is summarized in Table 1 below. The incidence of gastric cancer from the SEER database (>95% adenocarcinoma) was found to be declining both in the rural as well as urban population over the last 4 decades, however, the incidence remained higher in the rural population throughout this period (Supplementary Figure S1). On Kaplan–Meier analysis of survival estimates from the NCDB, unadjusted median OS for patients from RA was 12.3 months, and for those from MA was 13.3 months. OS rate (3-year, 5-year, and median) is summarized in Figure 1 below. Additionally, we saw similar trends of inferior disease-specific survival (DSS) using the SEER database (Supplementary Figure S2), where >95% gastric cancer patients had gastric adenocarcinoma.

On the univariate analyses, patients residing in RA, those with high grade (III/IV) disease, female gender, primary site location of cardia/fundus and uninsured, had a significantly worse OS, when compared to MA patients, low grade (I/II) disease, male gender, primary site location of body + lesser/greater curvature and insured patients respectively. These findings are summarized in supplementary Table S1. The multivariable Cox proportional hazards regression models showed that patients residing in RA, high grade (III/IV), uninsured patients, male gender, primary site at cardia and fundus, had a significantly worse OS when compared to patients residing in MA, those with low-grade tumors (I/II), insured patients, female gender, primary site at body of stomach or lesser/greater curvature of the stomach respectively. When comparing racial backgrounds, the Asian population did significantly better than the white population. Conversely, white patients did better when compared to black patients. These results are further summarized in Table 2 below. 

To assess timely management in these gastric adenocarcinoma patients, several variables were evaluated to examine the differences between RA and MA patient population. When looking at the median time (in days) of initiating any treatment from the time of diagnosis, it was observed that it was initiated significantly earlier in MA when compared to RA. These findings are summarized in Table 3 below. However, specifically surgery and radiation therapy were initiated in RA much earlier than MA. 

When assessing the quality of management among rural and metro regions, it was reflected that post-surgery the RA patients had a higher number of positive surgical margins when compared to MA (6.9% versus 6.3%; *p* < 0.01). Also, the 30-day and 90-day mortality post-surgery was significantly higher in RA patients compared to MA patients (within 30 days mortality—5.9% versus 4.9%; within 90 days mortality—11.8% versus 10%; *p* < 0.01). Though there is no established number of lymph nodes that need to be examined during gastrectomy, however, 15 is an acceptable standard. When comparing this proportion where 15 or more lymph nodes were examined surgically, it was found that 20% of MA patients underwent a procedure where 15 or more lymph nodes were examined in comparison to 19% in RA patients (*p* < 0.035). 

## 4. Discussion

Over the last few years, the incidence and mortality from gastric cancer have declined significantly, however, it is not clear whether these benefits are being dispersed equally across different geographic regions [11]. Our research in neuroendocrine tumors identified socio-demographic disparities in cancer outcomes [12]. Building upon our previous work, we further aimed to study disparities in the gastric cancer population. 

Gastric adenocarcinoma is the most common type of gastric cancer, followed by gastric lymphoma. Gastric adenocarcinoma is further characterized based on the location in the stomach, into cardia, and non-cardia cancers. Non-cardia cancers, found in the lower portion of the stomach, are associated with risk factors like chronic gastritis. Cancers involving cardia are associated with obesity, gastroesophageal reflux disease (GERD), and infection with Helicobacter pylori [4]. From a prognosis standpoint, cardia adenocarcinoma tend to have a worse prognosis compared to non-cardia cancers as demonstrated by several studies [13,14]. In this retrospective analysis, RA had a higher number of cardia adenocarcinoma versus non-cardia. In the multivariate model, the primary location of the tumor was found to influence survival with cardia cancers having worse overall outcomes compared to body or greater/lesser curvature primary location. The possible explanation includes higher incidence of metastasis and recurrences in cardia cancers [13].

When looking at gender and age-specific disparities among RA and MA, we found that the median age of patients diagnosed with gastric adenocarcinoma was lower in RA (68.7) compared to MA (69.5). In both regions, the incidence of GC in males was much higher than females (RA—71%, UA—67%). These findings are similar to what has been stated by Bray et al. in the study on global trends, which showed that the risk of developing gastric cancer is much higher in males than females. In fact, in developed countries, gastric cancer was more likely to be diagnosed in males than females, whereas in developing countries, the ratio was slightly lower [1]. This significant difference in gender disparity is unclear, but likely has a component of exposure in addition to genetics. Of note, we found that females had a slightly better OS compared to their male counterparts, as observed in several other population based studies [4,5,15].

We also observed differences in the racial composition of the MA and RA populations. MA had a larger proportion of African Americans and Asians, whereas RA had a larger proportion of native Americans. With regards to survival outcomes, the multivariable analysis revealed that Asian patients (HR 0.68; *p* < 0.01) had significantly better OS when compared to their white counterparts. Studies of gastric cancer have consistently shown survival benefits amongst the Asian population, and that is mainly attributed to early-stage and younger age at presentation [16,17,18]. Besides, Asian patients tend to have more distal cancers [19,20]. Distal tumors tend to cause early obstruction, resulting in early medical intervention.

There exists mixed evidence in regards to the survival data amongst AA patients, where some studies have reported AA performed poorly when compared to their white counterparts, while other studies have documented improvement in outcomes [16,21,22]. Poor outcomes could be attributed to environmental and genetic factors. It has been reported that AA patients tend to have higher rates of TP53 mutation, a universally accepted aggressive tumor marker, attributing to about 89% patients, in comparison to 40% in the Caucasian population [23]. Other contributing factors could be high prevalence of Helicobacter pylori infections seen in AA, and presentation at an older age [16,24,25,26]. However, the difference in the racial composition did not probably contribute to the observed differences in survival among MA and RA patients in our study, especially since the multivariable model adjusted for race.

In addition to the factors described above, diet has been strongly associated with both gastric cancer mortality and incidence. Ngoan et al. conducted a study in 13,000 Japanese subjects and found that high consumption of processed meat was associated with increased mortality in gastric cancer whereas that of green and yellow vegetables was related to a significant decline in mortality [27]. A cohort study in the Netherlands examined a total of 120,852 men and women, and reported that intake of salt and cured meat were positively associated with the risk of gastric cancer [28]. Similarly, the Hisamaya study reported a positive association between high dietary salt intake and the incidence of gastric cancer [29]. Therefore, it is well established that dietary patterns do play a major role in the development and outcomes of gastric cancer, but given the limited data provided in the NCDB, we could not assess the dietary patterns in different geographic regions. Interestingly, however, in a recent study, Euler et al. did not see any significant differences in the dietary intake by measures of rurality [30]. Therefore, we are unsure if dietary pattern influenced our observed differences in the outcomes between the rural and urban population. We think, however, that examining lifestyle and dietary differences in different geographic and ethnic populations and how they correlate with gastric cancer incidence and mortality, should be investigated further.

Multimodality therapies have proven to benefit GC patients; however, surgery stands as one of the most critical modalities in GC. As a result, the quality of surgery dictates survival [31]. The proportion of individuals who had >15 regional lymph nodes examined in MA (19.6%) was slightly higher than RA (18.7%). Studies have shown that the higher the number of lymph nodes examined is associated with longer disease-free survival in gastric cancer patients [32,33]. In a retrospective study, Deng et al. showed the number of dissected lymph nodes ≥15 was associated with better prognosis in lymph node-positive gastric cancer [32]. Also, it is a well-known fact that patients with positive margin disease tend to have a higher chance of recurrence when compared to patients with a negative margin resection. This ratio of positive margin post-surgery was noticed to be slightly higher among patients in RA when compared to MA (6.9% versus 6.3%; *p* < 0.01). Besides, higher 30 day and 90-day post-operative mortality were observed in RA when compared to MA (within 30 days mortality—5.9% versus 4.9%; within 90 days mortality—11.8% versus 10%; *p* < 0.01). These results speak in favor of surgeons in MA who are more specialized and tend to have more experience in operating complicated GC patients, further resulting in better outcomes. Timing of surgery post-diagnosis is another essential criterion, where our retrospective analysis showed that MA patients are operated at a median of 28 days, in comparison to 33 days for patients living in RA post-diagnosis. However, a study showed that neoadjuvant chemotherapy-surgery time interval does impact pathological response but does not impact survival [34]. Therefore, we are unsure whether this slight delay in surgery in RA patients had any influence on their survival. 

A significantly higher number of Individuals in MA (14.3%) are diagnosed with gastric cancer at an earlier stage (Stage I) when compared to RA (13.3%). These observations are likely secondary to limited access to health care and the availability of diagnostic equipment in RA. Studies evaluating outcomes of gastric cancer in China have shown that gastric cancers are usually diagnosed at a relatively advanced stage when it becomes symptomatic, and it is associated with a lower 5-year survival rate [35]. On the contrary, in Japan, it is diagnosed at earlier stages owing to their advanced diagnostic techniques; hence, higher survival rates are observed in Japan [36]. Likewise, in less developed areas in China, the mortality rate was much higher due to limited diagnostic techniques. This was demonstrated in a study by Li et al. when evaluating the overall burden of different cancers in rural and urban areas of China. The five-year OS of different cancers combined was much higher in urban than in rural areas. The five year OS of gastric cancer specifically was 32.65% in urban areas and 30.52% in rural areas [5]. Likewise, the mortality rate of all age groups was much higher in rural than in urban areas. Socioeconomic factors have a significant impact on the overall quality of care in rural and metropolitan areas. Access to tertiary care centers, advanced diagnostic equipment, and experienced providers are all factors that may explain the observed disparities in mortality and survival in rural and urban areas. In addition to limited access to quality care, the economic status of individuals in rural areas is also an essential factor that restricts their ability to afford the necessary diagnostic tests and treatment. In RA, the median household income of 95.5% of individuals was less than $63,000, whereas, in MA, this proportion was 65.8% (*p* < 0.01). In addition to income, previous research has shown that uninsured patients are likely to present with advanced-stage cancer of different types and less likely to receive definitive treatment [37]. 

Multivariable analysis revealed that insured patients tend to have better OS when compared to uninsured patients. When evaluating the proportion of insured individuals in UA and MA (93.8%), the analysis revealed same proportion of insured patients in both settings. Therefore, it is not possible to deduce disparities in survival benefits between insured and uninsured individuals in either UA or MA. Hence, more data is required to understand the relationship between insurance status and disease prognosis specific to gastric cancer. 

To our knowledge, this is the first population-based study investigating the rural–urban disparities in the care of patients with GC in the US utilizing the NCDB. Previous studies have been conducted in other Asian countries and one in the US, which evaluated patients from 2004 to 2009, utilizing the SEER registry. Using the NCDB, we evaluated the impact of different socio-demographic factors on the OS of patients residing in MA and RA. Besides, we were also able to examine different quality metrics associated with surgery. However, our study is not without limitations. Firstly, we had limited information on the stage of tumors as well as specific treatments in the NCDB. Secondly, being a retrospective study, it is not free from selection and misclassification bias, which may have impacted the outcomes. Thirdly, we tried to be consistent with most US studies in defining rural and urban areas; however, different definitions of rurality can cause misclassification bias [38,39]. 

## 5. Conclusions

Our NCDB-based study showed that there are significant socio-demographic disparities in the care of patients with GC. RA patients had marginally worse overall survival when compared to their MA counterparts. In addition, race, insurance status and sex were also associated with survival in the multivariable model. Access to healthcare, variations in patient care, environmental and lifestyle factors such as diet as well as genomic differences, are all potential factors that affect the OS. Our results are consistent with the available literature in GC, including studies demonstrating survival differences in different populations undergoing surgical treatment in the United States. These disparities are of concern and highlight where health policies need to be tailored to improve outcomes in all patients with GC across the U.S. 

## Figures and Tables

**Table 1:** Demographic and clinical characteristics of patients with gastric adenocarcinoma residing in rural and metro areas.

Variable		Rural	Metro	*p*-Value
Count, N (%)		12,365 (14)	75,881 (86)	
Age, median (range in years)		69 (40–90)	70 (40–90)	<0.01
Race	White (%)	10,739 (87)	57,053 (75)	<0.01
	Black (%)	1182 (10)	11,785 (16)	
	Asian (%)	119 (1)	5285 (7)	
Sex	Male (%)	8739 (71)	50,835 (67)	<0.01
	Female (%)	3626 (29)	25,046 (33)	
Primary Site	Cardia + fundus (%)	6174 (50)	30,480 (40)	<0.01
	Body + lesser/greater curvature (%)	395 (3)	2740 (4)	
	Antrum + pylorus (%)	658 (5)	5304 (7)	
	Stomach, NOS (%)	5138 (42)	37,357 (49)	
Staging	I (%)	1510 (27)	10,071 (29)	<0.01
	II (%)	1219 (21)	6968 (20)	
	III (%)	1439 (25)	8469 (24)	
	IV (%)	1527 (27)	9458 (27)	
Insurance	Not insured (%)	2918 (3.8)	411 (3.3)	<0.01
	Insured (%)	71,139 (93.8)	11,061 (93.8)	
	Unknown (%)	1824 (2.4)	353 (2.9)	

NOS—not otherwise specified.

**Table 2:** Multivariable Cox proportional hazards regression model highlighting OS in the gastric adenocarcinoma patient population.

Variable		Hazard Ratio	95% Confidence Interval (CI)	*p*-Value
Grade (III/IV) vs. grade (I/II)		1.49	1.47–1.52	<0.01
Charlson Comorbidity Score (≥1 vs. none)		1.06	1.04–1.08	<0.01
Primary location (body + lesser/greater curvature vs. cardia + fundus)		1.23	1.18–1.28	<0.01
Primary location (antrum + pylorus vs. cardia + fundus)		1.02	0.98–1.06	0.27
Race	Black vs. White	1.03	1.01–1.06	<0.01
	Asian vs. White	0.68	0.65–0.71	<0.01
Female vs. male		0.97	0.95–0.99	<0.01
Uninsured vs. insured		1.32	1.26–1.38	<0.01
Rural vs. urban		1.05	1.02–1.07	<0.01

**Table 3:** Median time to initiate therapy from the time of diagnosis of gastric adenocarcinoma (in days), in rural versus metro patient population.

Treatment Modality	Rural	Metro	*p*-Value
Chemotherapy	38	39	0.219
Surgery	33	28	<0.01
Radiation	46	54	<0.01
Any treatment	26	27	<0.01

## Supplementary Materials

The following are available online at https://www.mdpi.com/2072-6694/12/1/157/s1, Figure S1: Stomach adenocarcinoma incidence based on the SEER database from 1975 to 2016, Table S1: Univariate Cox proportional hazards regression model highlighting OS in the gastric adenocarcinoma patient population. Figure S2 Disease-specific survival of gastric cancer patients using SEER database (from 1975 to 2015).

## References

[1] Bray F., Ferlay J., Soerjomataram I., Siegel R.L., Torre L.A., Jemal A. (2018). Global cancer statistics 2018: GLOBOCAN estimates of incidence and mortality worldwide for 36 cancers in 185 countries. CA Cancer J. Clin..

[2] Siegel R.L., Miller K.D., Jemal A. (2019). Cancer statistics, 2019. CA Cancer J. Clin..

[3] Jim M.A., Pinheiro P.S., Carreira H., Espey D.K., Wiggins C.L., Weir H.K. (2017). Stomach cancer survival in the United States by race and stage (2001–2009): Findings from the CONCORD-2 study. Cancer.

[4] Rawla P., Barsouk A. (2019). Epidemiology of gastric cancer: Global trends, risk factors and prevention. Prz. Gastroenterol..

[5] Yang N., Hendifar A., Lenz C., Togawa K., Lenz F., Lurje G., Pohl A., Winder T., Ning Y., Groshen S. (2011). Survival of metastatic gastric cancer: Significance of age, sex and race/ethnicity. J. Gastrointest. Oncol..

[6] Wang J., Sun Y., Bertagnolli M.M. (2015). Comparison of Gastric Cancer Survival Between Caucasian and Asian Patients Treated in the United States: Results from the Surveillance Epidemiology and End Results (SEER) Database. Ann. Surg. Oncol..

[7] Strong V.E., Wu A.-W., Selby L.V., Gonen M., Hsu M., Song K.Y., Park C.H., Coit D.G., Ji J.-F., Brennan M.F. (2015). Differences in gastric cancer survival between the U.S. and China. J. Surg. Oncol..

[8] Li X., Deng Y., Tang W., Sun Q., Chen Y., Yang C., Yang F., Cao G., Ding Y., Zhao G. (2018). Urban-Rural Disparity in Cancer Incidence, Mortality, and Survivals in Shanghai, China, During 2002 and 2015. Front. Oncol..

[9] Glynn M.E., Keeton K.A., Gaffney S.H., Sahmel J. (2018). Ambient Asbestos Fiber Concentrations and Long-Term Trends in Pleural Mesothelioma Incidence between Urban and Rural Areas in the United States (1973–2012). Risk Anal..

[10] Paquette I., Finlayson S.R.G. (2007). Rural Versus Urban Colorectal and Lung Cancer Patients: Differences in Stage at Presentation. J. Am. Coll. Surg..

[11] Fontana E., Smyth E.C., Cunningham D., Rao S., Watkins D., Thompson J., Waddell T., Peckitt C., Chan I., Starling N. (2016). Improved survival in resected oesophageal and gastric adenocarcinomas over a decade: The Royal Marsden experience 2001–2010. Gastric Cancer.

[12] Gosain R., Ball S., Rana N.K., Groman A., Gage-Bouchard E., Dasari A., Mukherjee S. (2019). Geographic and demographic disparities in neuroendocrine tumors (NETs): A population-based study. J. Clin. Oncol..

[13] Chen W.Y., Cheng H.C., Wang J.D., Sheu B.S. (2013). Factors that affect life expectancy of patients with gastric adenocarcinoma. Clin. Gastroenterol. Hepatol..

[14] Ghidini M., Donida B.M., Totaro L., Ratti M., Pizzo C., Benzoni I., Lomiento D., Aldighieri F., Toppo L., Ranieri V. (2019). Prognostic factors associated with survival in a large cohort of gastric cancer patients resected over a decade at a single Italian center: The Cremona experience. Clin. Transl. Oncol..

[15] Cormedi M.C.V., Katayama M.L.H., Guindalini R.S.C., Faraj S.F., Folgueira M.A.A.K. (2018). Survival and prognosis of young adults with gastric cancer. Clinics.

[16] Kim J., Sun C.L., Mailey B., Prendergast C., Artinyan A., Bhatia S., Pigazzi A., EllenhorN J.D.I. (2009). Race and ethnicity correlate with survival in patients with gastric adenocarcinoma. Ann. Oncol..

[17] Merchant S.J., Li L., Kim J. (2014). Racial and ethnic disparities in gastric cancer outcomes: More important than surgical technique?. World J. Gastroenterol..

[18] Irino T., Takeuchi H., Terashima M., Wakai T., Kitagawa Y. (2017). Gastric Cancer in Asia: Unique Features and Management. Am. Soc. Clin. Oncol. Educ. B.

[19] DaCosta Byfield S.A., Earle C.C., Ayanian J.Z., McCarthy E.P. (2009). Treatment and outcomes of gastric cancer among United States-born and foreign-born Asians and Pacific Islanders. Cancer.

[20] Yu X., Hu F., Li C., Yao Q., Zhang H., Xue Y. (2018). Clinicopathologic characteristics and prognosis of proximal and distal gastric cancer. OncoTargets Ther..

[21] Howard J.H., Hiles J.M., Leung A.M., Stern S.L., Bilchik A.J. (2015). Race influences stage-specific survival in gastric cancer. Am. Surg..

[22] Bautista M.C., Jiang S.F., Armstrong M.A., Kakar S., Postlethwaite D., Li D. (2015). Significant Racial Disparities Exist in Noncardia Gastric Cancer Outcomes Among Kaiser Permanente’s Patient Population. Dig. Dis. Sci..

[23] Van Beek E.J.A.H., Hernandez J.M., Goldman D.A., Davis J.L., McLaughlin K., Ripley R.T., Kim T.S., Tang L.H., Hechtman J.F., Zheng J. (2018). Rates of TP53 Mutation are Significantly Elevated in African American Patients with Gastric Cancer. Ann. Surg. Oncol..

[24] Yao J.C., Tseng J.F., Worah S., Hess K.R., Mansfield P.F., Crane C.H., Schnirer I.I., Reddy S., Chiang S.S., Najam A. (2005). Clinicopathologic behavior of gastric adenocarcinoma in Hispanic patients: Analysis of a single institution’s experience over 15 years. J. Clin. Oncol..

[25] Al-Refaie W.B., Tseng J.F., Gay G., Patel-Parekh L., Mansfield P.F., Pisters P.W.T., Yao J.C. (2008). The impact of ethnicity on the presentation and prognosis of patients with gastric adenocarcinoma: Results from the national cancer data base. Cancer.

[26] Grad Y.H., Lipsitch M., Aiello A.E. (2012). Secular trends in helicobacter pylori seroprevalence in adults in the United States: Evidence for sustained race/ethnic disparities. Am. J. Epidemiol..

[27] Ngoan L.T., Mizoue T., Fujino Y., Tokui N., Yoshimura T. (2002). Dietary factors and stomach cancer mortality. Br. J. Cancer.

[28] Van Den Brandt P.A., Botterweck A.A.M., Goldbohm R.A. (2003). Salt intake, cured meat consumption, refrigerator use and stomach cancer incidence: A prospective cohort study (Netherlands). Cancer Causes Control..

[29] Shikata K., Kiyohara Y., Kubo M., Yonemoto K., Ninomiya T., Shirota T., Tanizaki Y., Doi Y., Tanaka K., Oishi Y. (2006). A prospective study of dietary salt intake and gastric cancer incidence in a defined Japanese population: The Hisayama study. Int. J. cancer.

[30] Euler R., Jimenez E.Y., Sanders S., Kuhlemeier A., Van Horn M.L., Cohen D., Gonzales-Pacheco D., Kong A.S. (2019). Rural-urban differences in baseline dietary intake and physical activity levels of adolescents. Prev. Chronic Dis..

[31] Choi Y.Y., Noh S.H., Cheong J.H. (2015). Evolution of gastric cancer treatment: From the golden age of surgery to an era of precision medicine. Yonsei Med. J..

[32] Deng J., Liang H., Sun D., Pan Y., Zhang R., Wang B., Zhan H. (2009). Outcome in relation to numbers of nodes harvested in lymph node-positive gastric cancer. Eur. J. Surg. Oncol..

[33] Hundahl S.A. (2002). Staging, stage migration, and patterns of spread in gastric cancer. Semin. Radiat. Oncol..

[34] Brenkman H., Van Putten M., Visser E., Verhoeven R., Nieuwenhuijzen G., Slingerland M., Van Hillegersberg R., Lemmens V., Ruurda J. (2018). Timing of postoperative chemotherapy in patients undergoing perioperative chemotherapy and gastrectomy for gastric cancer. Surg. Oncol..

[35] Zeng H., Chen W., Zheng R., Zhang S., Ji J.S., Zou X., Xia C., Sun K., Yang Z., Li H. (2018). Changing cancer survival in China during 2003–15: A pooled analysis of 17 population-based cancer registries. Lancet Glob. Health.

[36] Sitarz R., Skierucha M., Mielko J., A Offerhaus G.J., Maciejewski R., Polkowski W.P. (2018). Gastric cancer: Epidemiology, prevention, classification, and treatment. Cancer Manag. Res..

[37] Walker G.V., Grant S.R., Guadagnolo B.A., Hoffman K.E., Smith B.D., Koshy M., Allen P.K., Mahmood U. (2014). Disparities in stage at diagnosis, treatment, and survival in nonelderly adult patients with cancer according to insurance status. J. Clin. Oncol..

[38] Weissman S., Duffus W.A., Vyavaharkar M., Samantapudi A.V., Shull K.A., Stephens T.G., Chakraborty H. (2014). Defining the rural HIV epidemic: Correlations of 3 definitions-South Carolina, 2005–2011. J. Rural Health..

[39] Ratcliffe M., Burd C., Holder K., Fields A. (2016). Defining Rural at the U.S. Census Bureau.

