# Supplementary material for: Socio-Demographic Disparities in Gastric Adenocarcinoma: A Population-Based Study
Source: Cancers (Basel). 2020 Jan 9;12(1):157. doi: 10.3390/cancers12010157 (PMC7016781; doi:10.3390/cancers12010157)
Supplement: Supplementary file 1 [file cancers-12-00157-s001.pdf]

Supplementary Tables:

Supplementary Table 1: Univariate Cox proportional hazards regression model highlighting OS in the gastric adenocarcinoma patient population

Variable		Hazard ratio	95% Confidence Interval (CI)	p-value
Grade (III/IV) vs. grade (I/II)		1.43	1.40 – 1.45	<0.01
Charlson Comorbidity Score (≥ 1 vs. none)		1.11	1.09 – 1.13	<0.01
Primary location (body + lesser/greater curvature vs. cardia + fundus)		1.32	1.27 – 1.38	<0.01
Race	Black vs. White	1.03	1.01 – 1.06	<0.01
	Asian vs. White	0.66	0.64 – 0.69	<0.01
Female vs. male		1.05	1.03 – 1.07	<0.01
Uninsured vs. insured		1.08	1.03 – 1.13	<0.01
Rural vs. urban		1.08	1.05 – 1.10	<0.01

Supplementary Figures:

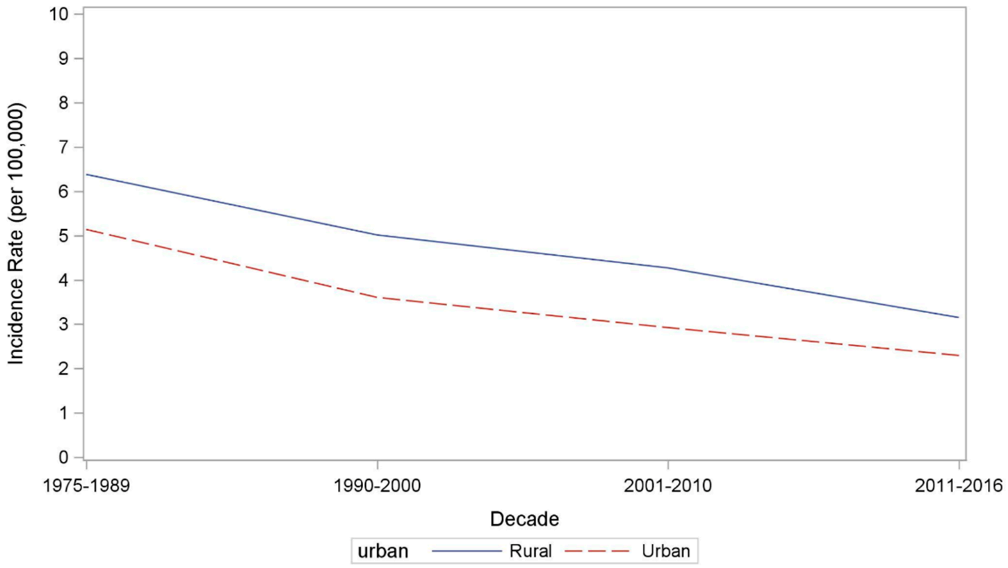

Supplementary Figure 1: Stomach adenocarcinoma incidence based on the SEER database from 1975 to 2016

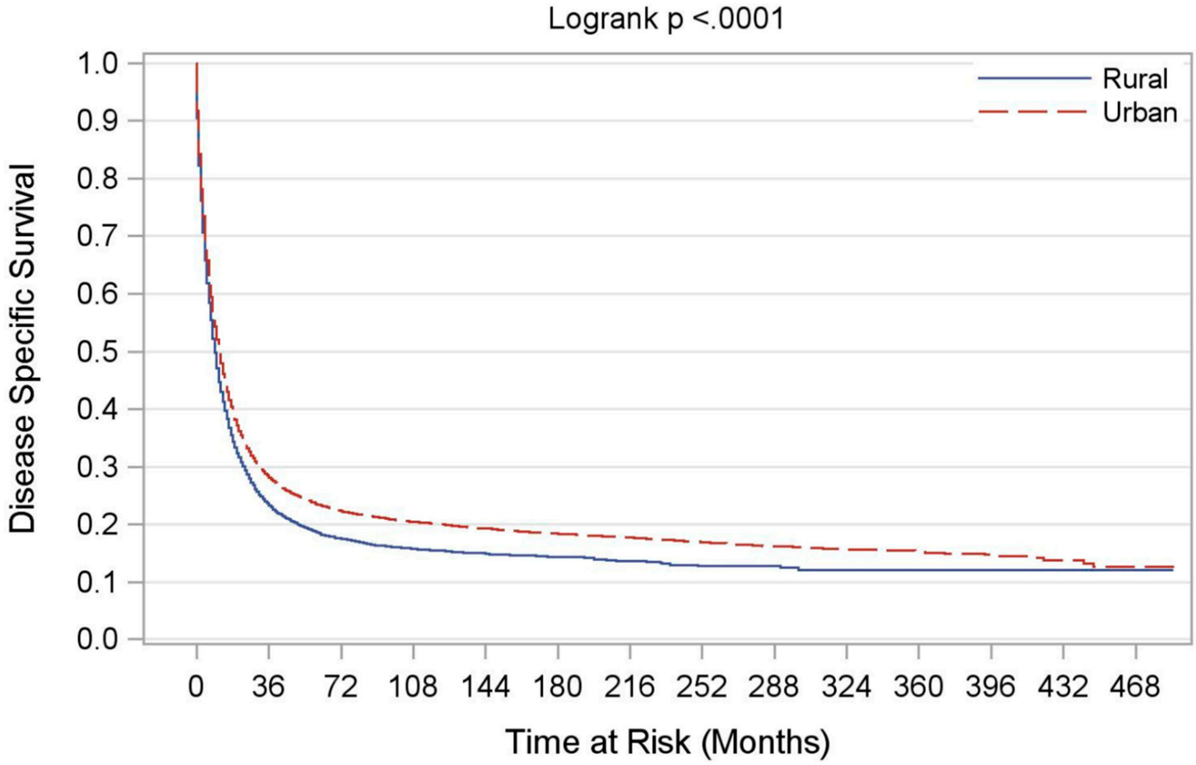

Strata	3-yr Surv.Rate (95% CI)	5-yr Surv.Rate (95% CI)
Total	0.28 (0.28, 0.28)	0.23 (0.23, 0.23)
Rural	0.24 (0.23, 0.25)	0.19 (0.18, 0.20)
Urban	0.28 (0.28, 0.29)	0.24 (0.23, 0.24)

Supplementary Figure 2: Disease-specific survival of gastric cancer patients using SEER database (from 1975 to 2015)
